# Activin Receptor Type IIB Inhibition Improves Muscle Phenotype and Function in a Mouse Model of Spinal Muscular Atrophy

**DOI:** 10.1371/journal.pone.0166803

**Published:** 2016-11-21

**Authors:** Min Liu, David W. Hammers, Elisabeth R. Barton, H. Lee Sweeney

**Affiliations:** 1 Department of Physiology, University of Pennsylvania, Philadelphia, Pennsylvania, United States of America; 2 Department of Pharmacology & Therapeutics, University of Florida College of Medicine, Gainesville, Florida, United States of America; 3 Myology Institute, University of Florida College of Medicine, Gainesville, Florida, United States of America; 4 Department of Applied Physiology and Kinesiology, College of Health and Human Performance, University of Florida, Gainesville, Florida, United States of America; Iowa State University, UNITED STATES

## Abstract

Spinal muscular atrophy (SMA) is a devastating neurodegenerative disorder that causes progressive muscle atrophy and weakness. Using adeno-associated virus-mediated gene transfer, we evaluated the potential to improve skeletal muscle weakness via systemic, postnatal inhibition of either myostatin or all signaling via the activin receptor type IIB (ActRIIB). After demonstrating elevated p-SMAD3 content and differential content of ActRIIB ligands, 4-week-old male C/C SMA model mice were treated intraperitoneally with 1x10^12^ genome copies of pseudotype 2/8 virus encoding a soluble form of the ActRIIB extracellular domain (sActRIIB) or protease-resistant myostatin propeptide (dnMstn) driven by a liver specific promoter. At 12 weeks of age, muscle mass and function were improved in treated C/C mice by both treatments, compared to controls. The fast fiber type muscles had a greater response to treatment than did slow muscles, and the greatest therapeutic effects were found with sActRIIB treatment. Myostatin/activin inhibition, however, did not rescue C/C mice from the reduction in motor unit numbers of the tibialis anterior muscle. Collectively, this study indicates that myostatin/activin inhibition represents a potential therapeutic strategy to increase muscle mass and strength, but not neuromuscular junction defects, in less severe forms of SMA.

## Introduction

Spinal muscular atrophy (SMA) is an autosomal recessive neurodegenerative disease characterized by loss of motor neurons in the anterior horn of the spinal cord. This disorder affects 1 in 11000 infants with a carrier frequency of 1 in 50 [1], resulting from reduced expression of survival motor neuron protein (SMN). The *SMN* gene is duplicated as an inverted repeat on each allele of human chromosome 5, typically existing as a telomeric copy of *SMN* (*SMN1)*, which is deleted or mutated in over 98% of SMA patients [2], and at least one copy of the centromeric *SMN* (*SMN2*), which provides the basis for survival in the absence of *SMN1* [3]. Alternate allelic variations have been described that include more than one *SMN1* and/or no *SMN2* copies, however [4]. On the basis of age of onset and severity of the symptoms, SMA can be subdivided into three clinical groups [5]. Acute type I form is characterized by severe progressive muscle weakness and hypotonia that is seen either at birth or within the first 6 months of life. Children with SMA type I will never be able to sit without support and usually die by the age of two years. Type II children are able to sit but cannot walk; and type III children are able to sit and walk with or without support. Type II and III patients generally have a milder progression and the potential for normal life expectancy.

SMA is characterized by profound muscle atrophy and weakness. This pathology results largely from denervation but also appears to have intrinsic muscle defects caused by SMN deficiency [6, 7]. For instance, myoblasts isolated from type I SMA patients display substantial impairments in cell proliferation, differentiation, and acetylcholine receptor aggregation [8], suggesting nerve-independent impairments in muscle development. Based on the genetic and clinical features in SMA, several directions for therapy development have been pursued. One strategy focuses on compensation for the disease symptoms, as opposed to directly targeting the genetic mutation. Attention has been devoted to treatments that can increase functional skeletal muscle mass by enhancing growth and/or preventing breakdown. This may be most important for the type II/III SMA subjects who suffer from persistent muscular deficits.

The activin family of ligands of the transforming growth factor (TGF)-β superfamily, which ligate and activate the activin receptor type IIB (ActRIIB; a TGFβ type II receptor), includes activins, growth differentiation factor (GDF) 11, and, most notably in the context of muscle, myostatin (Mstn) [9]. Mstn, in particular, has emerged as a potent negative regulator of skeletal muscle mass, as demonstrated by hypermuscularity caused by its inactivation in a variety of species [10]. Mstn is produced predominantly in skeletal muscle and is secreted as a latent precursor protein consisting of a mature C-terminal peptide dimer encased by the N-terminal, inhibitory pro-peptide. After cleavage of the pro-peptide by BMP-1/tolloid family proteases [11], the mature dimer is released to ligate ActRIIB, causing dimerization with ALK4/5 (TGFβ type I receptors), and subsequent activation of the SMAD2/3 signaling pathway [12]. Activin A and GDF11 also negatively affect skeletal muscle, causing atrophy [13] and inhibiting muscle differentiation [14, 15]. Due to these detrimental effects on muscle, blockade of ActRIIB ligands, thereby promoting muscle hypertrophy, is a potential therapy for neuromuscular disorders, such as SMA.

Among animal SMA models, two forms of transgenic lines have been primarily used to study the disease and facilitate drug development, *Smn*^*-/-*^; *SMN2*^*+/+*^ mouse lines, with mean life-spans of ~7 days [16, 17], and the *Smn*^*-/-*^; *SMN2*^*+/+*^;*SMN2Δ7*^*+/+*^ (often referred to as Δ7) mouse line, which survives 14–21 days [18, 19]. In these severe SMA mice, however, Mstn manipulation, whether by genetic inactivation or post-natal inhibition, does not improve the phenotype [20, 21]. This is understandable given the rapid loss of motor neurons and short lifespan associated with the severe phenotype. However, the possibility remains that an observable therapeutic benefit by activin inhibition may be possible in a milder form of SMA, with only partial motor neuron loss.

Less severe mouse models of SMA have recently been introduced, providing the same drug target but with a less severe baseline phenotype than the popular lines mentioned above [22]. One of these models, the SMA^C/C^ mouse (hereafter referred as the C/C mouse), harbors four relative copies of *SMN2* inserted into the *Smn* locus and demonstrates reduced Smn levels [22]. Phenotypically, this mouse line has a normal lifespan, exhibits reduced bodyweight and muscle mass compared to wild-type littermates, and displays mild impairment of motor unit activation and efficiency [22, 23]. Thus, this mouse line appears to represent a model of human type III SMA.

The aim of the present study was to evaluate the therapeutic potential of myostatin/activin inhibition on the phenotype of C/C mice. Herein, we report that young C/C mice display elevated SMAD3 activation and aberrant expression of ActRIIB ligands compared to wild-type littermates. Inhibition of ActRIIB activation by AAV-mediated systemic expression of the soluble ActRIIB (sActRIIB) [24] or the protease-resistant Mstn propeptide [25] improves both muscle mass and muscle function compared to control treated C/C mice. Thus myostatin/activin inhibitory strategies appear to have therapeutic potential for the treatment of mild cases of SMA.

## Results

**Fig 1:**
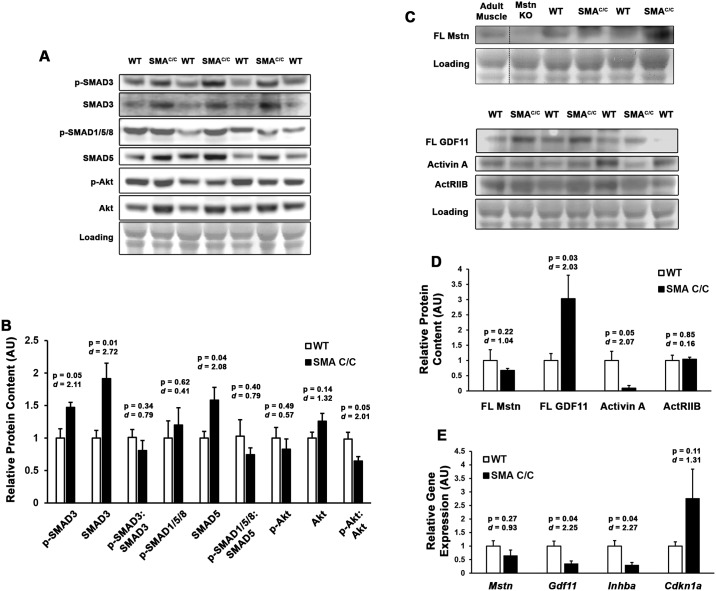
Hyperactivation of activin signaling pathway in young SMA^C/C^ mouse skeletal muscle. (**A-B**) Quadriceps muscles from 4 week-old SMA^c/c^ mice (n = 3) and wild-type (WT) littermates (n = 4) were immunoblotted for phosphorylated and total SMAD3, SMAD1/5/8, and Akt content. (**C-D**) Protein content of full-length (FL) myostatin (Mstn), FL GDF11, Activin A, and activin IIB receptor (ActRIIB) was measured by immunoblotting IgG-depleted quadriceps lystate samples. Immunoblotting quantifications are normalized to Ponceau Red-visualized loading. Adult muscle and Mstn knockout (KO) samples are included to demonstrate specificity of the antibody used. (**E**) Relative gene expression of *Mstn*, *Gdf11*, *Inhba*, and *Cdkn1a* (normalized to *Gapdh*) of the 4 week-old mouse quadriceps, as measured by real-time PCR. Data were analyzed using Student’s T-tests, and are presented as mean ± SEM with p-values and effect size (*d*) displayed.

Given that many muscle-centric studies of SMA, using either mouse or cell-culture models, have suggested that myoblast differentiation is affected by Smn deficiency [7, 26–28], and therefore suggesting an effect in postnatal muscle growth *in vivo*, we investigated if aberrant activin/SMAD activity was apparent in the skeletal muscle of 4 week-old C/C muscles. In comparison to WT quadriceps, C/C mice exhibited elevated p-SMAD3 and total SMAD3 content (Fig 1A and 1B), suggesting altered activin/SMAD dynamics. In agreement with this observation, the antagonistic p-SMAD1/5/8 and p-Akt pathways were unchanged. Interestingly, the muscle content of full-length Mstn and activin A were not elevated (the latter actually being decreased) in C/C mice (Fig 1C and 1D), suggesting these ligands are unlikely to be involved in the increased SMAD3 activity. Full-length GDF11, however, was elevated ~3-fold, and ActRIIB protein levels are unchanged by the disease. Gene expression of *Mstn*, *Gdf11*, and *Inhba* (gene for activin A) were all decreased in C/C muscle (Fig 1E), while *Cdkn1a* (gene for p21^Cip1^; a SMAD3 gene target) showed elevation, consistent with p-SMAD3 elevations. The observed disparity between GDF11 protein and *Gdf11* gene expression could indicate either inhibitory transcriptional feedback or reduced RNA stability in C/C muscles. These data support that ActRIIB hyperactivation is a feature of C/C muscle, and suggest activin inhibitory strategies may improve the C/C phenotype.

**Fig 2:**
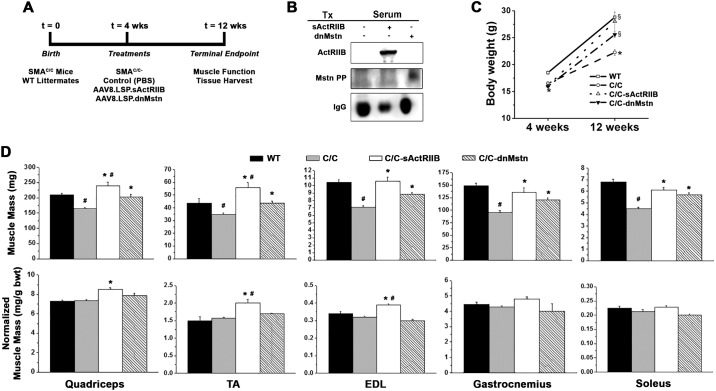
Activin signaling inhibition rescues muscle mass deficits in SMA^C/C^ mice. (**A**) Study design of the activin inhibition study, where 4 week-old SMA^c/c^ mice received control (PBS; n = 6), AAV8.LSP.sActRIIB (n = 6), or AAV8.LSP.dnMstn treatments (n = 6), and were evaluated in comparison to wild-type (WT; n = 6) littermates at 12 weeks of age. (**B**) Detection of systemic soluble ActRIIB (sActRIIB) or dominant-negative myostatin pro-peptide (dnMstn) in the serum of treated SMA^c/c^ using antibodies against ActRIIB and Mstn N-terminus (Mstn PP). Endogenous mouse IgG serves as a loading control. (**C**) Mouse body weights at 4 weeks and 12 weeks of age, displayed as mean ± SEM and analyzed using one-way ANOVA (Bonferroni-Dunn post-hoc tests; *p < 0.05 vs. WT values; §p < 0.05 vs. control C/C values). (**D**) Muscle masses of quadriceps, tibialis anterior (TA), extensor digitorum longus (EDL), gastrocnemius, and soleus muscles displayed as both absolute mass (top) and normalized to bodyweight (bottom) of WT and SMA^C/C^ treatments group mice at 12 weeks of age. Data are displayed as mean ± SEM and analyzed using one-way ANOVA (Bonferroni-Dunn post-hoc tests; ^#^p < 0.05 vs. WT values; *p < 0.05 vs. control C/C values).

To test this hypothesis, 4 week-old C/C mice were treated with PBS (Control), AAV8.LSP.sActRIIB (containing a soluble ActRIIB construct driven by a liver-specific promoter) [24], or AAV8.LSP.dnMstn (containing a protease-resistant form of the Mstn propeptide driven by a liver-specific promoter) [25], and were evaluated after 8 weeks of treatment (study depicted in Fig 2A). As shown in Fig 2B, both the sActRIIB and dnMstn transgenes were detectable in the serum of treated mice, resulting in significant increases in body mass, comparable to WT littermates (Fig 2C).

**Fig 3:**
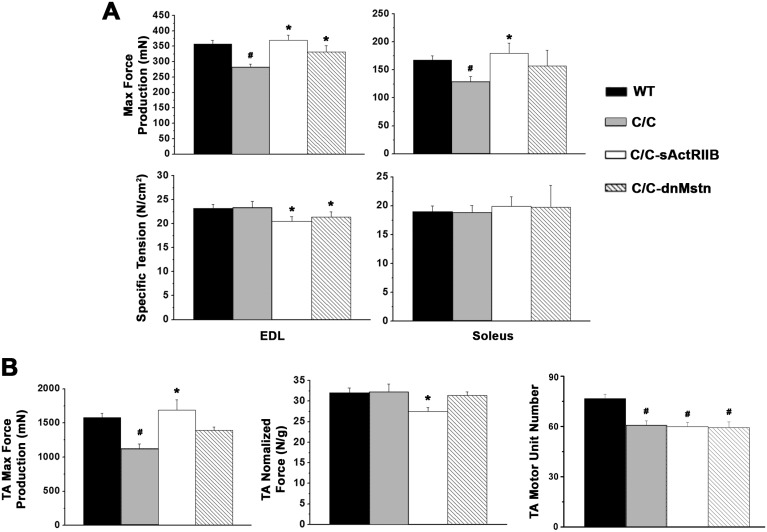
Activin inhibition improves contractile properties of SMA^C/C^ mice. (**A**) Muscle contractile properties of extensor digitorum longus (EDL; *in situ*) and soleus (*ex vivo*) muscles from 12 week-old wild-type (WT; n = 6) and SMA^C/C^ mice from control (C/C; n = 6), AAV8.LSP.sActRIIB (C/C-sActRIIB; n = 6), and AAV8.LSP.dnMstn (C/C-dnMstn; n = 6) treatment groups in terms of absolute peak tetanic isometric force production (top) and specific tension (bottom). (**B**) *In situ* contractile properties of the tibialis anterior (TA), in terms of absolute peak tetanic isometric force production (left) and mass-normalized force (middle). TA motor unit number was also evaluated (right). Data are displayed as mean ± SEM and analyzed using one-way ANOVA (Bonferroni-Dunn post-hoc tests; ^#^p < 0.05 vs. WT values; *p < 0.05 vs. control C/C values).

While control C/C mice demonstrated significantly lower absolute muscle mass than WT mice, both sActRIIB and dnMstn treatments were able to rescue the absolute mass of all muscles studied, while sActRIIB additionally rescued the bodyweight-normalized muscle mass (Fig 2D). The quadriceps and tibialis anterior (TA) masses of the sActRIIB treated group actually surpassed the values of the WT cohort. In agreement with the observed increases in muscle mass, the absolute force production of the extensor digitorum longus (EDL), soleus, and TA were improved by both activin inhibition strategies (Fig 3A and 3B), while normalized force values [whether by cross-sectional area (CSA) or muscle mass] were not improved. It is also important to note that neither treatment reversed motor unit number deficit of the C/C TA (Fig 3B **right**).

**Fig 4:**
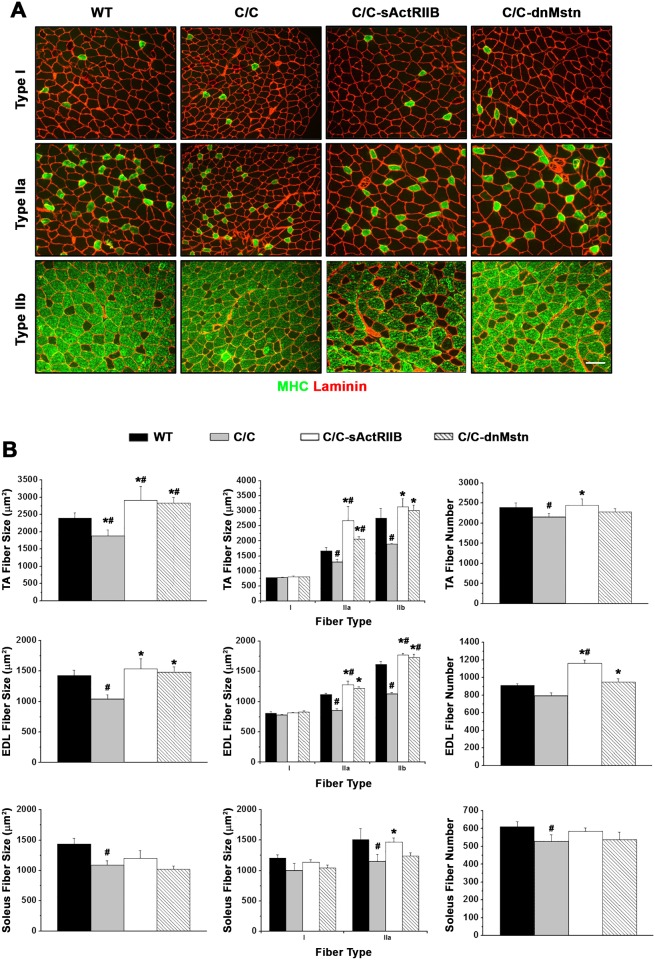
Fast twitch muscle fiber size of SMA^C/C^ mice rescued by activin inhibition. (**A**) Representative tibialis anterior (TA) muscle cross-sections stained with anti-myosin heavy chain (MHC) isoform and anti-laminin for fiber-typing and area analysis. Scale bar indicates 100μm. (**B**) Total muscle fiber size (left), muscle fiber size by fiber type (middle) and total muscle fibers (right) from the TA, extensor digitorum longus (EDL), and soleus muscles from 12 week-old wild-type (WT; n = 6) and SMA^C/C^ mice from control (C/C; n = 6), AAV8.LSP.sActRIIB (C/C-sActRIIB; n = 6), and AAV8.LSP.dnMstn (C/C-dnMstn; n = 6) treatment groups. Data are displayed as mean ± SEM and analyzed using one-way ANOVA (Bonferroni-Dunn post-hoc tests; ^#^p < 0.05 vs. WT values; *p < 0.05 vs. control C/C values).

As the pathological process may proceed differently in different muscle groups, muscle morphological analysis was performed on TA, EDL, and soleus muscles (Fig 4). TA and EDL muscles contain a large number of fast glycolytic fibers that express MHC type II and were selected as fast muscle type, whereas the soleus muscle contains a large proportion of slow oxidative fiber that express MHC type I and was chosen as a slow muscle type. We examined the fiber type distributions (based on myosin heavy chain expression) and found there were no significant differences in C/C muscles as compared to WT mice (data not shown). Interestingly, muscle atrophy in C/C mice was associated with both decreased muscle fiber size and fiber number (Fig 4B). Fiber CSAs in the TA, EDL and soleus muscle of C/C mice were significantly lower than those in WT mice, while the fiber number in C/C mice was reduced by ~11.0%, ~11.4%, and ~15.9% in TA, EDL and soleus muscles, respectively (Fig 4B **right**). Both sActRIIB and dnMstn treatments resulted in increased fiber CSA in TA and EDL, but not in the soleus. The hypertrophy associated with these treatments was due to increases in the size of fast fiber types (IIa and IIb), as well as increases in fiber numbers (in the TA and EDL). Interestingly, slow-oxidative (type I) fibers were not affected by the C/C atrophic phenotype.

**Fig 5:**
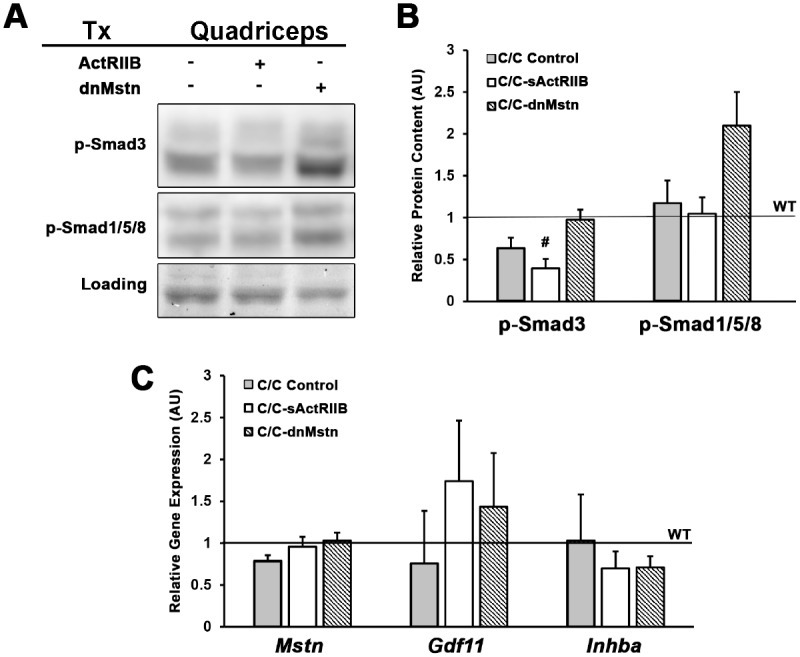
SMAD signaling altered by activin inhibition of SMA^C/C^ mice. (**A-B**) Phosphorylated levels of SMAD3 and SMAD1/5/8 in quadriceps lysates of 12 week-old SMA^C/C^ mice from control (C/C; n = 3), AAV8.LSP.sActRIIB (C/C-sActRIIB; n = 4), and AAV8.LSP.dnMstn (C/C-dnMstn; n = 4) treatment groups, normalized to Ponceau Red-visualized loading and relative to wild-type (WT) littermate levels. (**C**) Relative gene expression of *Mstn*, *Gdf11*, and *Inhba* (normalized to *Gapdh*) of 12 week-old SMA^C/C^ quadriceps, as measured by real-time PCR and displayed relative to WT levels. Data are displayed as mean ± SEM and analyzed using one-way ANOVA (Bonferroni-Dunn post-hoc tests; ^#^p < 0.05 vs. WT values; *p < 0.05 vs. control C/C values).

In quadriceps homogenates from this 12 week-old cohort, we found that the elevated p-SMAD3 levels of young C/C mice dissipated with age, and only the sActRIIB treatment further reduced SMAD3 activation (Fig 5A and 5B). Phosphorylated SMAD1/5/8 of dnMstn treated mice trended towards an increase in content. Additionally, it is important to note that though *Gdf11* trended towards increased gene expression in both treatment groups, activin inhibition did not appear to cause compensatory upregulation of ActRIIB ligands (Fig 5C).

## Discussion

SMA is a devastating neurodegenerative disorder that causes progressive muscle degeneration and weakness, affecting 1 in 11000 infants. It is one of the leading genetic causes of infant death in the world. Herein, we describe aberrant activin expression and signaling in young mice of the mild C/C SMA model. We also implement two approaches of activin inhibition to improve muscle force production in this less severe form of SMA via the soluble form of the extracellular domain of the ActRIIB (sActRIIB) or the protease-resistant Mstn propeptide (dnMstn). This study is the first demonstration of effective activin inhibition in a less-severe model of SMA. Both treatments increased absolute skeletal muscle mass in all hindlimb muscles studied, with concomitant increases in peak tetanic force. Importantly, while there was no rescue of the motor unit deficit, this treatment did not cause a further reduction in motor unit numbers in C/C mice.

Animal models of SMA have been used to study disease pathology and develop therapeutic interventions (see review in [29]). Mice have a single *Smn* gene that is similar to human *SMN1*. Since mutant embryos that lack a functional *Smn* gene die before birth [30], human SMN2 has been used as a disease modifier to recapitulate the genetic situation seen in SMA patients. In this study, we characterized the functional deficiencies in a milder mouse model, known as the C/C mouse, which has recently been developed by Regeneron in collaboration with the Jackson Laboratory and SMA Foundation [22]. Our characterization of this C/C SMA mouse line reveals that, at 12 weeks of age, the C/C mice showed ~21% lower body weight, 21–36% absolute muscle mass reduction, and a 21–29% decrease in muscle strength as compared to WT mice. Using histological analysis, we noted the muscle atrophy in C/C mice was associated with decreased muscle fiber size and fiber number. In addition, the specific force was not significantly different between WT and C/C mice, suggesting that the reduced force production can be attributed to the smaller muscle size and that muscle contractile strength is not significantly affected. To further examine the severity of disease pathology in this model, we studied the preservation of functional motor units in the TA. We used the same measure that has been performed to study therapeutic treatments in a murine model of amyotrophic lateral sclerosis (ALS) [31]. The number of motor units estimated by this method has been reported to be proportional to the motor neuron counts using histological staining [32]. In this investigation, we observed moderate decrease (~27%) in the number of functional motor units in the TA, which may be due to either motor unit loss or neuromuscular junction transmission failure. Collectively, this evidence suggests that C/C mice exhibit milder impairment of motor function compared to existing severe models, thus can be used as a model of less severe human disease (type III SMA) [33] and would be an appropriate model for developing drug compounds in SMA that target this patient population. This is important to emphasize about this model, as there are no approved drug treatments for patients with SMA. Although potential therapies are in trials [34], most strategies are focused on management of disease by preventing or treating the complications [35, 36].

In this study we found that in young (4 week-old) C/C mice, activin content and signaling is aberrant compared to WT littermates. Of specific interest, the observed elevations in p-SMAD3 content and gene expression of the SMAD3 target gene, *Cdkn1a*, coincide with a 3-fold increase in the ActRIIB ligand GDF11, which has ~92% homology with Mstn. Considering reports of the inhibitory effect of GDF11 on myogenesis [14, 15, 37], these data suggest dysregulation of ActRIIB signaling involving GDF11 may contribute to the SMA phenotype of C/C muscle. Indeed, inhibition of ActRIIB signaling via sActRIIB corrects many aspects of the C/C phenotype including rescuing of muscle mass and function to a greater extent than dnMstn treatment. This suggests that additional ligands than Mstn are contributing to the negative regulation of muscle growth, consistent with the observation of heightened GDF11 levels in the muscles of growing C/C mice.

Soluble activin receptors have been used to promote muscle growth in normal mice [38] and models of neuromuscular disorders [20, 24], and target a number of activin family members beyond myostatin. Injection of a sActRIIB extracellular domain led to a 60% increase in skeletal muscle mass in normal mice and in a mouse model of ALS by increasing skeletal muscle fiber size [39]. Similarly, administration of recombinant ActRIIB to caveolin deficient mice countered a portion of muscle atrophy [40]. Using the same approach in mdx mice, our laboratory has found ActRIIB blockade led to increased skeletal muscle mass and force production in the EDL [24]. In comparison to purely Mstn targeted therapies, the ActRIIB is able to inhibit additional ligands such as activin A/B [41], inhibin, and GDF11 [42] that signal through the ActRIIB and negatively regulate muscle mass [14], as well as BMP9. Modulation of multiple ligands that act through common signaling pathways may further improve muscle mass and function, but increases the risk of off-target side effects (e.g., [43]). Examination of activin family member expression did not differ in the C/C mice compared to WT mice after 8 weeks of treatment, suggesting that the potential for off-target effects remains in this model for SMA. Thus, given the modest strength increase following sActRIIB expression as compared to myostatin inhibition alone, myostatin inhibition likely would deliver considerable benefit with fewer possible off-target toxicities. Specific inhibition of GDF11 may also represent a method to limit off-target effects, as the above data suggests this ligand is involved in the atrophic phenotype.

In contrast, sActRIIB has been reported to not be beneficial in severe SMA mice, as its administration to Δ7 SMA mice on the second day after birth showed minimal improvement in motor function and no extension of survival. This is likely due to the fact that the weakness and muscle loss is driven primarily by motor neuron loss and is not addressable by targeting only skeletal muscle. Indeed, our current treatments did not alter the motor neuron reduction found in C/C mice, suggesting that a combined strategy targeting both skeletal muscle and neuronal pathology could improve strength to a greater extent.

We carried out histological analysis and found the increased muscle mass in our study resulted from both hypertrophy and increased numbers of muscle fibers. Our quantification showed that, following Mstn inhibition, the average fiber CSA was 16–21% and 22–27% larger in TA and EDL respectively and the total fiber number was 11–16% higher in treated C/C mice (Fig 4). Mstn has been suggested to play two distinct roles in regulating muscle fiber number and size. A number of experiments have shown that the development of hyperplasia versus hypertrophy depends on the method to block Mstn activity and on the timing at which Mstn activity is lost [44–46]. Similar to this study, mice with a deletion of Mstn and transgenic mice expressing a dominant-negative form of ActRIIB showed increases in both fiber number and size [47]. In contrast, hypertrophy without hyperplasia has been reported in adult mice that were treated with antibodies to Mstn [46]. For example, muscle growth following postnatal Mstn blockade in *mdx* mice is entirely due to hypertrophy of myofibers [24]. Typically, the hyperplasia associated with Mstn deficiency is attributed to loss of regulation in pre/perinatal development, while postnatal inhibition promotes only hypertrophy of the existing myofibers. Therefore, one plausible explanation for the observed both hyperplasia and hypertrophy in the treatment groups is that the C/C mice may exhibit delayed development, thus are still undergoing developmental stages at time of treatment. Unlike the *mdx* mice, muscular deficits in SMA mice are caused by reduced levels of Smn. SMN is ubiquitously expressed in all cells and developmentally regulated in different tissues. With low levels of Smn expression, the mice might show delayed development [48]. Furthermore, the early loss of motor neurons in the C/C mice appears to result in a slight loss of fibers (Fig 4), which myostatin inhibition may somehow overcome, resulting in increased numbers of fibers.

In this study, we observed a differential response to activin inhibition by fiber type, as the fast muscles appear to respond more robustly to treatment than slow muscle. Though all treated muscles are significantly greater in mass than those of untreated C/C mice, the greatest increase in muscle mass was noted in the TA and the least in soleus. In addition, our histological analysis for the fiber CSA showed that activin inhibition caused significant increases solely in fast fiber type, IIa and IIb. Similar findings have been reported in Mstn knockout mice, in the studies of Mstn inhibition in the mdx mice [24, 25, 49], and also with Mstn inhibition in dystrophin-deficient dogs [45]. The mechanism of the reduced responses in the slow muscle is likely due to the differential expression of the ActRIIB in slow vs. fast fibers. The ActRIIB content in the EDL muscle has been reported to be approximately two fold greater than soleus muscle [49]. Therefore, fast muscles show greater response to activin-based interventions than slow muscles. Since we saw this same effect in dystrophic dogs [50], it likely will be recapitulated in humans.

The increased muscle mass appears to be functional, although somewhat less so in the case of the sActRIIB where the specific forces in TA and EDL are significantly lower in treated C/C mice (Fig 3). The EDL specific force was also decreased with the propeptide inhibition of Mstn. Nonetheless, all treated muscles displayed increased absolute force production. Decreased specific force has been reported as a consequence of Mstn inhibition, possibly as a result of hypertrophy without proportional incorporation of additional nuclei from satellite cells in fast fiber types [51]. Another possible explanation is that, when the muscle CSA increases, the muscle pennation angle changes as well. It is estimated that the Mstn deficient mice have a 38% greater pennation angle in EDL and 17% greater in soleus muscle [49]. The transmission of the muscle fibers to muscle is proportional to the cosine of pennation angle. As the muscle CSA increases, the net force transferred from each muscle fiber to entire muscle will decrease.

In the case of SMA, myostatin/activin inhibition does not address the underlying genetic defect, therefore it was not clear if solely increasing muscle mass by inhibiting ActRIIB signaling would be beneficial. Our data suggests that myostatin/activin inhibition could be utilized to augment correction of the primary defect by promoting rapid gains in muscle function. It may even be beneficial as a mono-therapy in less severe forms of SMA. While we considered the possibility that increasing muscle mass would increase the burden on the neuromuscular junctions and motor neurons, we found no differences in functional TA motor units between treated and untreated SMA mice. Thus two months of treatment did not affect motor neuron survival in C/C mice. Interestingly, Li *et al*. reported, using the non-invasive measure of compound muscle action potential (CMAP), that the C/C mouse does not decline in synchronized motor unit activation over several months of observation, yet have significantly less motor unit transmission than wild-type animals [23]. This evidence suggests the loss of functional motor units occurs during developmental stages and is not progressive in this model.

Collectively, systemic inhibition of ActRIIB signaling led to an increased skeletal muscle mass/size in all hindlimb muscles studied, with concomitant increases in peak tetanic force. The fast muscles, TA and EDL, respond more robustly to treatment than slow muscle (soleus). Although longer-term studies are necessary to better assess the potential impact on motor neuron survival, this study indicates that activin inhibition is an effective therapeutic strategy to enhance muscle function in a mouse model of SMA. Since this treatment does not alter the motor unit number in SMA mice, additional therapies that target motor neuron survival are needed to stabilize the innervated muscle mass. Thus, myostatin inhibition-based therapy can be an important potential therapeutic option for mild forms of the disease such as type II/III SMA, and even in more severe forms, if augmenting therapies that increase SMN expression are implemented.

## Materials and Methods

### Mice

All animal procedures were approved by and conducted in accordance to the University of Pennsylvania IACUC. Heterozygote breeding pairs were initially purchased from Jackson Laboratories (Stock # 8604). Mice were bred as heterozygous pairs and genotyped, as described by Osborne et al. [22]. Homozygous C/C mice were also distinguished by severe necrosis of the tail and ears, confirming genotyping results. Male C/C homozygous mice and wild-type (WT) littermate controls were used for this study. Mouse cohorts were euthanized at 4 weeks of age (biochemical analysis; N = 7) or at 12 weeks of age (viral treatment cohorts; N = 24).

### Vector production

AAV8.LSP.sActRIIB and AAV8.LSP.dnMstat virus stocks were produced by the University of Pennsylvania Vector Core as previously described [24, 25]. Briefly, the myostatin gene C terminal region from nucleotide 825–1131 of the reading frame was deleted via PCR. A D76A mutation was introduced by splicing overlap extension PCR. This results in a peptide resistant to proteolytic activation. The mutant transgene was named dominant negative myostatin (dnMstat) and was then cloned into an AAV transfer vector with a liver specific promoter, alpha1-antitrypsin promoter with ApoE enhancer (LSP), provided by Dr. Katherine High [38]. To produce a soluble form of the extracellular domain of the receptor IIB, a fusion construct of the mouse myostatin signal sequence, the extracellular domain of the activin IIB receptor, and the fixed chain region of IgG2a, was subcloned into the same AAV transfer vector.

### Viral injection

Four-week-old male C/C mice were injected intraperitoneally (i.p.) in the left lower quadrant of the abdomen with 300 μL of sterile PBS (control; n = 6), 1x10^12^ genome copies (gc) of AAV8.LSP.sActRIIB (n = 6), or 1x10^12^ gc of AAV8.LSP.dnMstn (n = 6) virus (diluted in 300 μl of sterile PBS). A cohort of WT littermates mice (n = 6) served as non-disease controls. Functional testing and tissue harvest were performed at 12 weeks of age.

### Muscle functional analysis

*In situ* muscle function was examined on tibialis anterior (TA) and extensor digitorum longus (EDL), while ex vivo functional testing was performed on isolated soleus muscle. Mice were deeply anesthetized via i.p. injection of ketamine-xylazine (80 and 10 mg/kg) and monitored throughout the experiment. For each preparation, the distal tendon of the TA and EDL were carefully dissected and individually tied with 4.0 braided surgical silk. The sciatic nerve was exposed and all of its branches were cut except for the common peroneal nerve. The foot was secured to a platform and the knee immobilized using a stainless steel pin, while being careful not to interfere with the blood supply to the muscles. The body temperature was monitored and maintained at 37°C.

The suture from the TA and EDL tendon was individually attached to the lever arm of a 305B dual-mode servomotor transducer (Aurora Scientific, Ontario, Canada). Muscle contractions were then elicited by stimulating the distal part of the sciatic via bipolar electrodes, using supramaximal square-wave pulses of 0.2 msec (701A stimulator; Aurora Scientific). Data acquisition and control of the servomotor were conducted using a Lab-View-based DMC program (version 5.202; Aurora Scientific). Optimal muscle length (L_o_) was determined by incrementally stretching the muscle until the maximum isometric twitch force was achieved. Three maximum isometric tetanic forces (Po) were acquired using a train of 150Hz supramaximal electrical pulses for 500 msec at the optimal length in the muscles and highest Po was recorded. A 2-minute resting period was allowed between each tetanic contraction. L_o_ was measured using digital calipers. The EDL specific forces (N/cm^2^) were calculated by dividing P_o_ by muscle CSA. Muscle CSA was estimated using the following formulae: muscle weight (g)/ [[(*L*_o_ (cm) x 0.45] x 1.06 (g/cm^3^)]. The TA relative force (N/g) was normalized by dividing Po by the muscle weight.

The TA motor unit number was measured by a series of isometric twitch contractions with stimuli of increasing intensity. The stimulus amplitude was initially set to 0 V and manually increased over a range of 10 V, which resulted in discrete increments in twitch force due to the successive recruitment of motor units. With each increment in force, the stimulus amplitude was held at constant voltage for at least 5 stimuli and then increased manually to elicit a larger twitch. This procedure was repeated until there was no further increase in force, indicating that all motor units were recruited. The number of step-wise increments of force was counted in a blinded fashion and taken as an estimate of the number of motor units in the TA muscles. This is a reliable and reproducible method that has been used to assess motor unit survival in both normal and mutant SOD1 mice [31].

Following *in situ* testing, the soleus muscle was subjected to isolated mechanical measurements using a previously described apparatus (Aurora Scientific, Ontario, Canada). Briefly, the soleus muscle was removed and placed in a bath of Ringer solution gas equilibrated with 95% O_2_/5% CO_2_. After determining L_o_ by supramaximal twitch stimulation, maximum tetanic forces were determined using 80Hz supramaximal electrical pulses for 1 sec at the optimal length. Stimulation was delivered via two parallel platinum electrodes that were positioned along the length of the muscle. The soleus CSA was estimated using the following formula:

$$\text{CSA }=\text{muscle weight }\left(\text{g}\right)/\;[[L_{\text{o}}\left(\text{cm}\right)\text{ x }0.\text{69}]\text{ x 1}.0\text{6 }(\text{g}/{\text{cm}}^{\text{3}})].$$

At the end of the mechanical measurements, the quadriceps, gastrocnemius, TA, EDL, and soleus muscles were removed from the animal, weighed, and snap-frozen in liquid nitrogen (biochemical analysis) or embedded in OCT and frozen in melting isopentane (histological analysis).

### Muscle morphological measures

Muscle morphological measures were performed on TA, EDL and soleus muscles. Transverse muscle sections (10 μm thick) were cut from the mid-belly of the muscles and mounted serially on gelatin-coated glass slides. Immunohistochemistry was performed on serial sections to determine the fiber sizes, fiber number, and myosin heavy chain (MHC) composition of the examined muscles, as described previously [52]. Rabbit anti-laminin (Neomarker, Labvision, Fremont, CA) was used to outline the muscle fibers for CSA quantification. The MHC antibodies used to determine the MHC composition of selected muscles were type I (BA-F8) at 1:50, type IIA (SC-71) at 1:10, and type IIB (BF-F3) at 1:3. Sections were blocked in 5% bovine serum albumin/phosphate-buffered saline (BSA/PBS) and incubated overnight in 5% BSA/PBS containing rabbit anti-laminin monoclonal antibody diluted 1:100 and an MHC primary antibody at the dilutions described earlier. Following washes in PBS, sections were incubated in appropriate secondary antibodies (Invitrogen, Carlsbad, California) for 1 hour in the dark at room temperature. Slides were washed and mounted with VectaShield with DAPI. All images were captured and processed on a fluorescence microscope (Leitz DMRBE; Leica, Bannockburn, Illinois) equipped with a digital camera system (MicroMAX; Princeton Instruments, Trenton, New Jersey). The entire section for each muscle was examined for quantification of fiber size and number using Imaging software (OpenLab; Improvision, Waltham, Massachusetts). The muscle fiber CSA and total fiber number of each muscle studied were determined.

### Immunoblotting

Snap-frozen quadriceps samples were finely crushed and homogenized in T-PER buffer (Thermo Scientific; Waltham, MA) supplemented with protease and phosphatase inhibititors (Thermo Scientific). Protein concentration of resulting supernatant was determined using Bio-Rad Protein Assay (Bio-Rad; Hercules, CA). Whole serum samples were diluted 1:3 in PBS. Samples were boiled in 4X sample buffer, subjected to SDS-PAGE using 4–12% SDS-polyacrylamide gels (Life Technologies; Grand Island, NY), and transferred to nitrocellulose membranes using the iBlot system (Life Technologies). Membranes were blocked in 5% milk-TBST and incubated with anti-ActRIIB (1:500; Sigma-Aldrich), myostatin (N-terminal; 1:1000; kind gift from Dr. Se-Jin Lee), p-SMAD3 (Ser423/425; 1:300; Cell Signaling), p-SMAD1/5/8 (Ser436/435; 1:300; Cell Signaling), SMAD3 (1:500; Cell Signaling), SMAD5 (1:500; Cell Signaling), p-Akt (Ser473; 1:500; Cell Signaling), Akt (1:1000; Cell Signaling), GDF11 (1:1000; R&D), myostatin (C-terminal; 1:1000; kind gift from Regeneron Pharmaceuticals), or activin A (1:1000; R&D) overnight at 4°C. Following TBST washes, membranes were incubated in the appropriate HRP-conjugated secondary antibody for 1 hour at room temperature, washed, incubated for 5 minutes in ECL reagent (Thermo Scientific), and imaged using the LI-COR C-DiGit (LI-COR Biosciences; Lincoln, NE) imaging system. Membranes were stained with Ponceau Red and imaged for loading normalization. Band signal intensities were measured using Image Studio Lite software (LI-COR Biosciences), normalized to sample loading, and reported relative to respective control samples. Specificity of Mstn and GDF11 antibodies were tested and verified using overexpression and knock-out models. Samples destined for probing with mouse monoclonal antibodies were pretreated with protein A/G-coated agarose beads (Santa Cruz Biotechnology) to remove endogenous IgGs prior to sample preparation.

### Real-time PCR

RNA was isolated from finely crushed snap-frozen mouse quadriceps samples using Trizol Reagent (Life Technologies), treated with DNAse (Promega; Madison, WI), and reverse transcribed using the SuperScript III kit (Life Technologies). Resulting cDNA was subjected to real-time PCR using RQG SYBR Green supermix (Qiagen) in a Rotor Gene Q real-time PCR machine (Qiagen) with the following mouse-specific primers: *Mstn* (forward) 5’- GAC CCG TCA AGA CTC CTA CAA CAG-3’ and (reverse) 5’- ATC GCA GTC AAG CCC AAA GTC TCT-3’; *Gdf11* (forward) 5’- GGC CGG CGT CAC ATC CGT ATC-3’ and (reverse) 5’- GCC GGA GCA GTA GTT GGC CT-3’; *Inhba* (forward) 5’- CAG GAG GGC CGA AAT GAA TGA ACT-3’ and (reverse) 5’- CCG CAC GTC CAG GGA ACT CTT T-3’; *Cdkn1a* (forward) 5’- GCT CAT GGC GGG CTG TC-3’ and (reverse) 5’- CTG CGC TTG GAG TGA TAG AAA T-3’. Primers for *Gapdh* [(forward) 5’-AGC AGG CAT CTG AGG GCC CA-3’ and (reverse) 5’-TGT TGG GGG CCG AGT TGG GA-3’] were used for ΔΔCt normalization.

### Data analysis

Data are expressed as mean ± standard error of mean (SEM), and analyzed using Student’s T-tests with effect size reported as Cohen’s *d* (*d*) or one-way ANOVA (Bonferroni-Dunn post-hoc tests; α = 0.05).
